# Real‐time ethics engagement in biomedical research: Ethics from bench to bedside

**DOI:** 10.15252/embr.201949919

**Published:** 2020-01-15

**Authors:** Jeremy Sugarman, Annelien L Bredenoord

**Affiliations:** ^1^ Johns Hopkins Baltimore MD USA; ^2^ University Medical Center Utrecht Utrecht the Netherlands

**Keywords:** S&S: Economics & Business, S&S: History & Philosophy of Science

## Abstract

Biomedical research often raises ethical questions that are usually addressed *ad hoc* or in retrospective. Real‐time ethical engagement as part of research may be better suited to identify ethical issues.

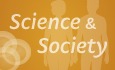

Biomedical research inevitably involves ethical issues. Some raise broad public concerns, particularly when researchers obviously violate established ethical norms. For example, He Jiankui's work using CRISPR/Cas9 to genetically modify human embryos to prevent HIV transmission, which resulted in the birth of the world's first two gene‐edited babies, generated widespread condemnation of this use of human germline modifications. Ethical issues also arise in the earlier phases of basic research, such as the public release of the HeLa cell genome by the European Molecular Biological Laboratory that created controversy over privacy concerns. At other times, ethical issues are more subtle and may not be recognized as such or raise public concern. For instance, there are important ethical considerations related to using banked biospecimens in translational research. Similarly, creating neurological chimeric mouse models involves moral considerations related to the potential humanization of these models 1, and embryo models from human stem cells are entangled in debates about the moral status of the embryo 2.

Nonetheless, efforts should be taken to identify and manage ethical issues as early as possible in order to provide ethical guidance throughout the entire research process, and mitigate negative effects, harms and wrongs (K.R. Jongsma & A.L. Bredenoord, under review). In this paper, we describe how ethics expertise can contribute to biomedical research through real‐time engagement and some of the challenges associated with such efforts. To do so, we offer our experiences with two particular examples: organoid technology and umbilical cord blood (UCB) banking and transplantation.

… efforts should be taken to identify and manage ethical issues as early as possible in order to provide ethical guidance throughout the entire research process…

## Is there an ethicist in the laboratory?

What exactly constitutes (bio)ethics expertise is far from a settled issue even though the issues have been broadly discussed in clinical settings 3. Nonetheless, some distinctive elements can be identified, such as skills in reasoning about morally relevant concepts and arguments, and an understanding of relevant literature and precedents. Regardless, ethics expertise can contribute to research policies and practices in several ways. First, ethicists can help to identify and raise awareness about whether ethical challenges are involved in particular research efforts. Second, they can alert scientists to relevant guidelines or scholarship. Third, they can provide normative judgments and deliberate about appropriate courses of action or the development of novel treatments and technologies. Fourth, they can help anticipate societal impact. Fifth, they can employ methods from the social sciences to conduct empirical bioethics research with, for example, end users (patients, professionals, data subjects), to inform an anticipatory and constructively guiding approach to research practices (K.R. Jongsma & A.L. Bredenoord, under review).

Just as in clinical settings, ethics engagement can take different forms. *Ad hoc* consultation—the “beeper ethicist”—is a familiar way of involving ethicists in clinical practice, such as whether to discontinue life‐sustaining interventions. *Ad hoc* consultations are also employed for ethical questions in basic and translational research. This approach may be incredibly valuable and sufficient for some research, but a single consultation may miss the potential for more added value of what ethicists can contribute.

At the other side of the spectrum is having an ethicist or ethics team work alongside 4 or embedded with the research team. This can vary from “desk experiments” where ethicists work with teams of basic and translational researchers, to robust, real‐time engagement that involves normative research and obtaining empirical data to help inform deliberations and decision‐making (K.R. Jongsma & A.L. Bredenoord, under review). To ground this discussion in real examples, we describe our experience with real‐time ethics engagement that has employed both conceptual–normative and empirical aspects to address organoid technology and UCB banking and transplantation.

## Organoid technology

Organoids are three‐dimensional self‐organized tissue cultures derived from stem cells. Organoids can be studied as models for organ growth and development, to test medications, or for transplantation. For example, gut organoids are used in the context of precision medicine for cystic fibrosis (CF), brain organoids can be used to study the biological aspects of psychiatric conditions, liver organoids are being developed for transplantation, and gastruloids that resemble early‐stage embryos provide insights into early embryonic development 5.

Of particular relevance are concerns about conflicts of interest if ethicists merely provide window dressing for what others might consider to be unethical research.

At an early stage, pioneers in organoid research recognized that while organoids have enormous scientific and clinical potential, there were some associated ethical issues, so they approached the University Medical Center Utrecht ethics team for advice 5. A small interdisciplinary team subsequently analyzed the ethics and research implications of organoid technology throughout the research cycle, from fundamental preclinical research to translational and clinical applications, as well as the societal impacts 5. The team also looked at the ethics of organoid biobanking. Internal funding made it possible to have a small team of ethicists embedded in both the Hubrecht Institute where the organoids were developed and the Wilhelmina Children's Hospital whose patients participated in organoid‐related research.

Organoid technology is particularly promising for CF, as it offers a strikingly accurate personalized model of the disease. Intestinal organoids, derived from rectal biopsy material, permit the prediction of individual drug response 6, but the value and concerns regarding this emerging technology were initially unclear. The team therefore decided to explore patients’ perspectives on organoid technology in a qualitative study. Specifically, the team conducted 23 interviews with 26 respondents: 14 adult patients and 12 parents of young patients with CF 6. In addition, the team conducted three focus groups with patients or parents of patients with metabolic disorders, to discuss the ethical challenges of a first in human liver organoid transplantation trial. These empirical studies provided invaluable input about what patients and tissue donors perceive as the key ethical dimensions of organoid technology.

Cystic fibrosis patients and their parents, for example, expressed an ambiguous relationship to organoids as both closely and distantly related to themselves. These and other findings inspired further reflection on the moral status of organoids and other key ethical themes such as commercial use, consent, and governance. Particularly, the notion of organoids as hybrids that relate to persons and their bodies and to technologies and markets in ambiguous ways helped to prompt rethinking about the (commercial) use and exchange of organoids in an ethically sound way 7.

The ethical challenges of organoid research are not limited to national borders, as legislation regarding the derivation, use, and storage of stem cells and the launch of clinical trials can be different on national, European Union (EU), and international levels. At the moment, the Utrecht ethics group is involved in an EU H2020 project aimed at building a European Organoid Biobank for patients with rare CF mutations (http://www.hitcf.org), where the ethics team co‐produces the governance and ethics of this biobank.

## Umbilical cord blood banking and transplantation

Umbilical cord blood banking is now commonplace, and UCB transplantation is a standard treatment option for a variety of diseases and conditions. However, as these practices were first being explored, an array of ethical challenges were encountered. Relatively soon after the first successful UCB transplantation was performed and the first UCB banks were being constructed, one of the scientific leaders sought ethics expertise, which initially resulted in a collaborative conceptual scoping paper, outlining some of the main ethical issues 8.

As described in more detail elsewhere 9, a series of ethics activities followed. First, in order to include the perspectives of additional stakeholders, a working group was assembled to deliberate the relevant issues and to offer guidance. Second, given that the informational needs and perspectives of pregnant women regarding the possible collection of UCB were unclear, a series of focus groups was conducted that proved invaluable in designing and implementing a recruitment and informed consent process for a public UCB bank. Third, in order to assess the effectiveness of these processes, a quantitative survey of those who donated UCB was performed. Fourth, qualitative analyses of marketing messages of different cord blood banks were carried out to inform deliberations about their ethical appropriateness. Subsequently, *ad hoc* consultations have been used to address emerging issues in UCB transplantation, for example, as it is being explored for non‐malignant conditions.

## Potential barriers

Despite the benefits, robust ethics engagement can face a series of potential barriers. For example, scientists must be open to ethics engagement and inquiry, and that requires collaboration, openness, time, and resources. It may also include the need for funding, which may not be trivial; however, in our experience, it accounts for only a small fraction of the funding for most scientific endeavors. When there are insufficient funds available to support ethics engagement, it can be possible to seek institutional or external support. Yet, the process of obtaining funding may take considerable time, slowing down the analyses of the ethical issues as research is proceeding.

There are options for engaging ethics expertise into basic and translational research in ways that can be grounded in the realities of scientific research.

Of course, ethics engagement can itself be associated with ethics concerns. Of particular relevance are concerns about conflicts of interest if ethicists merely provide window dressing for what others might consider to be unethical research. As with conflicts of interests associated with research in general, the nature of support and contributions should be transparent in describing the work and any resulting guidance, including publications and presentations. Depending on the nature of the initiative, it can be prudent to somehow involve those who are otherwise external to the project or an institution in developing or reviewing recommendations and publications. Additional ethics concerns arise when conducting empirical bioethics research, which can usually be addressed through existing oversight mechanism, such as review (or review exemption) by research ethics committees.

**Further reading**

**Boers SL, Van Delden JJM, Clevers H, Bredenoord AL (2016) Organoid biobanking: identifying the ethics.** ***EMBO Reports*** **17(7):938–41**

This paper reviews whether and to what extent organoids give new twists to the ethical challenges in stem cell research and related fields, particularly the ethical challenges related to the donation, storage, and use of organoids.

**Boers SN, Bredenoord AL (2018) Consent for governance in the ethical use of organoids.** ***Nature Cell Biology*** **20:642–645**

The authors propose consent for governance as a promising paradigm for the derivation, storage, and use of complex human tissue products, among which organoids. Consent for governance entails an initial consent procedure that provides donors with information on governance and shifts the ethical emphasis from initial consent to ongoing governance obligations, which include protection of donor privacy, participant engagement, benefit sharing, and ethical oversight.

**Bruce CR, Peña A, Kusin BB, Allen NG, Smith ML, Majumder MA (2014) An embedded model for ethics consultation: characteristics, outcomes, and challenges. *AJOB Empirical Bioethics* 5:3, 8–18,** https://doi.org/10.1080/23294515.2014.889775

In this paper, the authors describe a model of clinical ethics consultation, which they term “embedded ethics” and involves embedding clinical ethics consultants within clinical specialties and subspecialties based on institutional needs and areas of clinical ethicists’ expertise.

**Huch M, Gehart H, van Boxtel R, Hamer K, Blokzijl F, Verstegen MM, Ellis E, van Wenum E, Fuchs SA, de Ligt J, van de Wetering M, Sasaki N, Boers SJ, Kemperman H, de Jonge J, IJzermans JN, Nieuwenhuis EE, Hoekstra R, Strom S, Vries RR, van der Laan LJ, Cuppen E, Clevers H (2015) Long‐term culture of genome‐stable bipotent stem cells from adult human liver.** ***Cell*** **160(1–2):299–312**

This report describes that single mouse Lgr5^+^ liver stem cells can be expanded as epithelial organoids *in vitro* and can be differentiated into functional hepatocytes in vitro and in vivo. The authors also delineate conditions allowing long‐term expansion of adult bile duct‐derived bipotent progenitor cells from human liver.

**Porter KM, Danis M, Taylor HA, Cho, MK, Wilfond BS, on behalf of the Clinical Research Ethics Consultation Collaborative Repository Group (2018) The emergence of clinical research ethics consultation: insights from a national collaborative.** ***The American Journal of Bioethics*** **18:1, 39–45,** https://doi.org/10.1080/15265161.2017.1401156

The report describes a national research ethics consultation (REC) service as a forum in which to discuss challenging or novel ethical issues not fully addressed by regulations. This is a reaction to the increasing complexity of human subjects research and its oversight.

**Travis J. Privacy flap forces withdrawal of DNA data on cancer cell line.** ***Science*** **2013 March 26. Available at:** https://www.sciencemag.org/news/2013/03/privacy-flap-forces-withdrawal-dna-data-cancer-cell-line

This paper describes the controversy after the European Molecular Biology Laboratory (EMBL) announced that a research team had deciphered much of the genetic sequence of the widely used HeLa cell line and had made the information available publicly. EMBL has withdrawn those data and apologized for potentially violating the privacy of Henrietta Lacks, the woman who was the original source of the cells, and that of her descendants.

## Concluding comments

There are options for engaging ethics expertise into basic and translational research in ways that can be grounded in the realities of scientific research. While we have provided some examples based on our experience, more rich descriptions of both good and bad experiences with ethics engagement are needed to help inform the refinement of these approaches. Such information should be useful in establishing best practices for ethics engagement to improve the process of science. This seems far superior to retrospective ethics critiques once work is published. More importantly, engaging ethics expertise should enhance the possibility of conducting ethically sound research.

## Conflict of interest

Jeremy Sugarman is a member of Merck KGaA's Bioethics Advisory Panel and Stem Cell Research Oversight Committee; is a member of IQVIA's Ethics Advisory Panel; and has been a consultant to Portola Pharmaceuticals, Inc. Annelien Bredenoord is a member of IQVIA's Ethics Advisory Panel.
